# Supplementary material for: The influence of social determinants of health on orthopedic trauma outcomes in the United States: A scoping review
Source: PLOS Glob Public Health. 2025 Dec 29;5(12):e0005573. doi: 10.1371/journal.pgph.0005573 (PMC12747403; doi:10.1371/journal.pgph.0005573)
Supplement: Attachment — Submitted filename: Response to Reviewers.docx [file pgph.0005573.s004.docx]

Andreas K Demetriades, MBBChir, MPhil, FRCSEd, FEBNS

Academic Editor

PLOS Global Public Health

November 17, 2025

Dear Dr. Demetriades and Editorial Team:

Thank you for inviting us to submit a revised draft of our manuscript entitled **“The Influence of Social Determinants of Health on Orthopedic Trauma Outcomes in the United States: A Scoping Review”** to **PLOS Global Public Health**. We sincerely appreciate the time and effort that you and all three editors, as well as the reviewers, dedicated to providing thoughtful and constructive feedback.

It is with great pleasure that we resubmit our article for further consideration. We have carefully revised the manuscript to reflect the detailed suggestions you have offered, and we hope that these changes—along with the responses provided below—satisfactorily address all comments and concerns raised.

To facilitate your review of our revisions, we provide below a point-by-point response to the questions and comments outlined in your letter dated **October 14**. For clarity, we present each editor or reviewer comment in italics, followed by our author response in regular text, and the corresponding changes made in the manuscript.

**Reviewer #1:**

*Introduction:
The authors are not explicit about how limited awareness of research opportunities for researchers of certain backgrounds leads to under-representation of people of those backgrounds in studies. I assume the authors are alluding to these researchers being more keen to perform studies that affect people from their community and also more likely to get buy-in and recruitment.*

Thank you for these insightful comments. We agree that the Introduction did not clearly articulate that limited awareness of research opportunities among researchers from underrepresented backgrounds contributes to the under-representation of their communities in orthopedic trauma research. To clarify this relationship, we revised the Introduction to explain that structural barriers restricting research training, funding, and mentorship for individuals from historically marginalized backgrounds result in a less diverse research workforce.

We have added text to the Introduction to make this connection explicit (see revised lines 73-86 of the updated manuscript).

*There is no referencing of the multiple assertions made in the introduction between SDOH domains and the orthopaedic trauma outcomes. Please find references to support your views*

Thank you for noticing this oversight on our part. The appropriate references were added to the paragraph in lines 111-115.  *Methodology:
Why were distal humerus (unless this is what elbow meant), radial, and ulnar fractures not included for these limb fractures? I note that no spinal fractures, hand fractures or foot fractures were included. How do the authors define orthopaedic trauma?*

In this scoping review, we focused on major appendicular and pelvic fractures as the definition of orthopedic trauma. This does not include spinal, hand, or foot fractures, which all have their own subspecialty. Also, distal humerus, radial, and ulnar fractures are included, but this was not written in a clear way. Thank you for pointing out that the way the methods section was written was confusing. We have updated the Methods section in lines 187-191. *Results:
Table 2 content could be column 3 of table 1
Table 3 could be an additional column for table 1
Table 4 could be column 6 of table 1*

Thank you for the suggestion. Tables 2-4 were added to the final row of Table 1. I agree that decreased number of tables is better for the overall reader experience.

*It’s unclear why lower extremity fractures are separate from hip fractures, as I suspect you’re using hips to mean proximal femur fractures*

We thank you for this important point. Hip fractures, defined as proximal femur fractures (including femoral neck and intertrochanteric fractures), were considered separately from other lower extremity fractures because they differ in patient population, mechanism of injury, and clinical management. Hip fractures predominantly occur in older adults due to low-energy mechanisms such as falls related to frailty and osteoporosis, whereas other lower extremity fractures occur across a broader age range and result from both high- and low- energy trauma. Additionally, hip fractures have distinct surgical interventions, rehabilitation protocols, complication profiles, and mortality risks. For these reasons, we categorized hip fractures separately.  *It’s unclear how the qualitative data collected as per the methodology has been synthesized*

We thank the reviewer for highlighting this point. As this is a scoping review, our aim was to map the existing literature on orthopedic trauma outcomes and social determinants of health, rather than perform a formal qualitative synthesis. Qualitative data were summarized descriptively, with key study characteristics, SDOH factors, and outcomes categorized and reported using counts or percentages where applicable. This approach allowed us to present an overview of trends and gaps in the literature without combining or interpreting qualitative findings across studies. We have clarified this in the Methods section to avoid confusion in lines 214-217.

*The results do not seem to relate SDOH domains with outcomes, which was the thrust of the rationale for performing this study.*

We thank the reviewer for this important observation. As this is a scoping review, our primary aim was to **map the existing literature** rather than perform quantitative or formal qualitative analyses linking SDOH domains to outcomes. Due to the large amount of heterogeneous data—including differences in study populations, injury types, outcome measures, and SDOH variables—it was not possible to systematically relate each SDOH domain with specific outcomes. Instead, we presented the findings in **Table 1** to summarize what data are currently available and have been studied, providing a foundation to **identify gaps and guide future research** that can more rigorously examine these relationships. The previous edits made in lines 214-217 have clarified this rationale.

**Reviewer #2: Anh Nguyen**

*Thank you for your effort to conduct a timely and well-executed scoping review examining the influence of social determinants of health on orthopedic trauma outcomes in the United States. I would like give some recommendations:
1. It might be helpful to elaborate on the framework for SDOH and trauma outcomes that lead to your search strategy to make sure it is comprehensive.*

We thank the reviewer for their time reviewing the manuscript and suggestions. Our search strategy was guided by the CDC framework for social determinants of health, which includes five domains: healthcare access and quality, neighborhood and built environment, economic stability, education access and quality, and social and community context. Search terms were mapped to these domains and relevant orthopedic trauma outcomes to ensure a comprehensive capture of the literature. We have clarified this rationale in the Methods section lines 149-152.

*2. It might be helpful to mention key domains/words of SDOH and trauma outcomes in the search strategy section of the main text. I mean a summary of S2 file.*

We thank the reviewer for this suggestion. The four key concepts were added to the Methods section in lines 152-154.

*3. Please elaborate why and how your restrict your review scope to "the United States"*

The review was restricted to US-based studies to ensure consistency in healthcare systems, sociocultural contexts, and structural determinants that can influence orthopedic trauma outcomes. Restricting the review to U.S. studies ensures that the findings are not influenced by differences between countries, allowing for a more accurate mapping of the existing literature and identification of gaps relevant to U.S. populations. Lines 152-154 that were added to the Methods section clarify that US was one of the domains in the search strategy. Additional rationale was added to the Introduction in lines 120-126.

*4. It might be helpful to report on key findings across studies on "The Influence of Social Determinants of Health on Orthopedic Trauma Outcomes in the United States". Right now, it seems you are just counting the frequencies of SDOH and trauma outcomes across studies. How about the combination of these two?*

We thank the reviewer for this suggestion. While combining specific SDOH domains with individual trauma outcomes would provide additional insight, the large heterogeneity across the 40 included studies, including differences in study design, SDOH variables measured, outcome definitions, and statistical methods, precludes a meaningful synthesis of these combinations in the current scoping review. To maintain clarity and comprehensiveness, we have focused on summarizing the frequencies of SDOH domains and trauma outcomes separately, as presented in Tables 1-4. This approach allows readers to understand the scope of the existing literature and identify gaps, while providing a foundation for future research that could explore more detailed SDOH and outcome associations.

**Reviewer #3: Parker Frankiewicz**

*Overall, this manuscript adds clarity on consideration for the social determinants of health in orthopedic trauma literature and is structured well with JBI methodology for scoping reviews and PRISMA-ScR reporting guidelines. However, the manuscript would benefit from several revisions to strengthen transparency, interpretation, and methodological clarity.*

*Major Comments
1. Protocol Registration.
The manuscript notes that no protocol was registered. While not strictly required for scoping reviews, protocol registration (e.g., with Open Science Framework as PROSPERO does not permit registration of scoping reviews) enhances transparency and credibility. The authors workaround this adequately by uploading their full search strategy as supplemental information. However, the authors should also more explicitly explain the reasoning behind their decision to not register their initial protocol.

Recommendation: Acknowledge this limitation explicitly in the methods or discussion and explain why a protocol was not registered.*

We thank the reviewer for this suggestion. We agree that not registering a protocol is a limitation of this work, and if the project were initiated anew, we would have registered it to enhance transparency and credibility. While a formal protocol was not registered, we have provided the full search strategy as supplemental material (S2 File), ensuring reproducibility of our methodology. We have also added this as a limitation in the final paragraph of the discussion in lines 346-349.

*2. Clarification of Statistical/Methodological Approach.
The manuscript frequently reports proportions of studies that “referenced” a given SDOH domain (e.g., 80% included social/community context, 7.5% included education). However, it is not entirely clear what constitutes a “reference.” For example: Was an SDOH domain counted if mentioned in baseline demographics only? Or did the study need to analyze the SDOH factor as a predictor of outcomes? Were studies weighted equally regardless of sample size?

Recommendation: Provide more explicit criteria for how studies were coded as including a particular SDOH domain. Clarify whether this reflects analysis of SDOH as a variable in statistical analysis, mention in the text, or categorization of the study’s primary aim. This distinction is critical for interpretation, as a mere mention in baseline demographic data is not equivalent to rigorous SDOH analysis.*

We thank the reviewer for this important point. To clarify, we only counted an SDOH domain if it was explicitly included in the analysis of outcomes, not merely reported as part of baseline demographics or mentioned in passing. Each study was weighted equally regardless of sample size, as only the percentages and counts of each SDOH domain were used in the analysis.

We have updated the Methods section in lines 193-195 and 214-217 to make these criteria explicit, ensuring that the reported proportions reflect studies that analyzed SDOH as predictors or variables associated with outcomes rather than simple descriptive reporting.

*3. Study Quality Considerations
While quality assessment is not required in scoping reviews, the predominance of retrospective and single-center studies (70% and 52.5%, respectively) as reported in this analysis substantially limits generalizability.

Recommendation: Add a short subsection in the discussion that comments on methodological limitations of the included literature (e.g., retrospective design, lack of standardized SDOH data collection, reliance on chart review variables such as insurance or race). Even in the absence of a formal assessment of bias and quality of each source, the data the authors have reported regarding study type merits mention in the limitations.*

We thank the reviewer for this suggestion. We agree that the predominance of retrospective and single-center studies, as well as the variability in SDOH data collection and reliance on chart-based variables such as insurance or race, limits the generalizability of the findings. We have added a discussion about the limitations based on study quality to lines 340-345.

*4. Outcome Categorization
Table 7 reports that 67.5% of studies included outcomes in the category “other,” which obscures important heterogeneity especially given that this category is the largest of the table.

Recommendation: Consider listing the specific outcomes included under “other” whether in an appendix to the table or in the text of the results section itself. This would allow readers to fully appreciate the breadth of reported measures.*

We agree that the “other” category being the largest category limits the importance of the scoping review. We had tried to combat this in the original submission by including all outcomes categorized as “other” in Table 1 with the associated studies. We agree that this information may be hidden due to the amount of information in Table 1. We have added a clarifying sentence to the Results section in lines 277-278.  *5. Interpretation and Clinical/Policy Implications
The conclusions appropriately emphasize research gaps, but the implications for current clinical practice and health policy are underdeveloped. For example: How could trauma centers use SDOH data in discharge planning? How might insurance status and neighborhood deprivation guide targeted follow-up or resource allocation?

Recommendation: Expand discussion on practical applications of current evidence, even if limited, to strengthen relevance for clinicians and policymakers.*

We agree that expanding on the potential clinical and policy applications of integrating SDOH into orthopedic trauma care would strengthen the relevance of our findings. We have added a section to the discussion outlining how trauma centers could incorporate SDOH into discharge planning, follow-up coordination, and resource allocation, as well as ways policymakers might leverage these data to reduce disparities in trauma outcomes. Added section is seen in lines 326-335.

*6. U.S.-Specific Context
Because the review is limited to U.S. studies, it would be useful to highlight how U.S. health system characteristics (insurance-based access, trauma system organization) shape findings and limit generalizability internationally.

Recommendation: Elaborate on the US-specific context in both the limitation and discussion sections to ensure acknowledgement of limited generalizability as well as make the discussion section more relevant for US-based readers and future research.*

We appreciate the reviewer’s thoughtful comment regarding the US-specific context of this review. We have expanded both the Introduction (lines 120-126 and Discussion (349-352 to clarify how these characteristics of the US healthcare system shape the findings of the scoping review.  *Minor Comments
1. Abstract – Strengthen the concluding sentence by explicitly stating the main implication (e.g., “highlighting the need for standardized SDOH data collection and greater focus on non-surgical trauma care”).*

We have strengthened the concluding sentence of the abstract to explicitly highlight the main implication of our findings. This revision found in lines 63-65 clarifies the practical significance of the review for both researchers and clinicians.

*2. Tables – The authors could consider combining tables 5 and 6 (injury location and treatment overview) into a unifying figure or condensing to improve readability.*

We agree that the number of tables makes the prior submission difficult in terms of readability. Tables 2-4 were added to the final row of Table 1, as suggested by another reviewer.

*3. Terminology – Race and ethnicity are categorized under “social and community context,” but this may benefit from clarification. These variables encompass both a demographic categorization as well as systemic inequities; precise terminology would strengthen the manuscript.*

Thank you for pointing out that this was not explicitly stated in the paper. We have added lines 100-101 to clarify the categorization and importance.

Again, thank you for giving us the opportunity to strengthen our manuscript with your valuable comments and queries. We have worked hard to incorporate your feedback and hope that these revisions persuade you to accept our submission.

Sincerely,

Rachel Rumana

Corresponding Author

University of Miami Miller School of Medicine

Rxr1239@med.miami.edu
